# Risk factors for bile leakage after primary closure following laparoscopic common bile duct exploration: a retrospective cohort study

**DOI:** 10.1186/s12893-016-0201-y

**Published:** 2017-01-05

**Authors:** Dongbin Liu, Feng Cao, Jiafeng Liu, Dahua Xu, Yuehua Wang, Fei Li

**Affiliations:** Department of General Surgery, Xuanwu Hospital, Capital Medical University, Beijing, 100053 People’s Republic of China

**Keywords:** Laparoscopic common bile duct exploration, Primary closure, Bile leakage

## Abstract

**Background:**

Primary closure following laparoscopic common bile duct exploration (LCBDE) has been widely adopted because of the efficacy and safety in treatment of common bile duct (CBD) stones. However, the risk factors for bile leakage, the most common complication after primary closure, has not been clarified yet.

**Methods:**

A retrospective cohort study of patients who underwent LCBDE with primary closure after choledochotomy between Feb. 2012 and Jun. 2016 was performed. Risk factors for bile leakage were identified by logistic regression inculding demographic factors, preoperative condition and surgical details.

**Results:**

Between Feb. 2012 and Jun. 2016, a total of 265 LCBDE procedures were applied in our hospital and 141 patients with primary closure were included in this study. Bile leakage occurred in 11.3% (16/141) of these patients, and happened more frequently in patients with slender CBD (<1 *vs* ≥1 cm, 31.6% *vs* 7.0%, *p =* 0.04) and those managed by inexperienced surgeons (initial 70 cases *vs* later cases, 17.1% *vs* 5.6%, *p =* 0.04). After multivariable regression, the diameter of CBD [OR 95% CI, 3.799 (1.081–13.349), *p =* 0.04] and experience of surgeons [OR 95% CI, 4.228 (1.330–13.438), *p =* 0.03] were significantly related to bile leakage.

**Conclusion:**

Slender CBD and inexperienced surgeons were the high risk factors for bile leakage after primary closure following LCBDE.

## Background

Common bile duct (CBD) stone is the strong indication for surgical therapy, especially in patients with obstructive jaundice. With the development of laparoscopic equipment and technology, laparoscopic common bile duct exploration (LCBDE) has been widely used in clinical practice [1–4]. Many previous studies have demonstrated that LCBDE is safe and effective in treatment of CBD stone [2, 4, 5]. After LCBDE, primary closure or T-tube drainage will be applied according to the condition of CBD and experience of surgeon. In 1991, Phillip first reported the technique of LCBDE and T-tube drainage in treatment of CBD calculi encountered during laparoscopic cholecystectomy. On the basis of the experience of open CBD exploration, T-tube drainage has been widely adopted in the past two decades. However, T-tube drainage has many problems, such as fluid and electrolyte disturbance, sepsis, premature dislodgement, bile leakage, prolonged biliary fistula, late bile duct stricture, and possible peritonitis after removal of the T-tube, which accounted for 15% of all patients [6]. These complications and the need of satisfactory follow-up cholangiography prolonged the hospital stay and increased hospital expenses [7].

Primary duct closure after open CBD exploration was first described by Halstead as early as 1917. Since then, the debate between primary closure and T-tube drainage continued even in the era of laparoscopic surgery. In the past decade, numerous studies comparing primary with T-tube were published and revealed the feasibility and safety of primary closure [8–10]. Meta-analysis form Yin demonstrated that among patients with laparoscopic choledochotomy for CBD stones, primary closure of the CBD alone is superior to T-tube drainage in terms of postoperative and biliary-specific complications [11]. However, primary closure following LCBDE is still a technical demanding operation and with a relative high risk of bile leakage. Regardless of the widely use of primary closure after LCBDE, the risk factors for bile leakage is still largely unknown. Therefore, we performed this retrospective study to identify the risk factors for bile leakage after primary closure following LCBDE.

## Methods

### Patients selection

Patients with CBD stones who were admitted to our center between Feb. 2012 and Jun. 2016 were screened and those who received primary closure after LCBDE were included in this study. In our institution, the indications for primary closure following LCBDE are as follows: (1) CBD stones are confirmed by preoperative MRCP or CT with no intrahepatic bile duct stone; (2) The diameter of CBD is more than 0.8 cm; (3) No obvious inflammatory changes of CBD are detected intraoperatively; (4) No. of stones is less than 5; (5) Function of Oddi sphincter is normal without residual stone which is confirmed by intraoperative flexible choledochoscope. Patients with previous biliary surgery history or converted to open surgery were excluded from this study. This study was approved by the Ethics Committee of Xuanwu hospital.

### Operative techniques

**Fig. 1:**
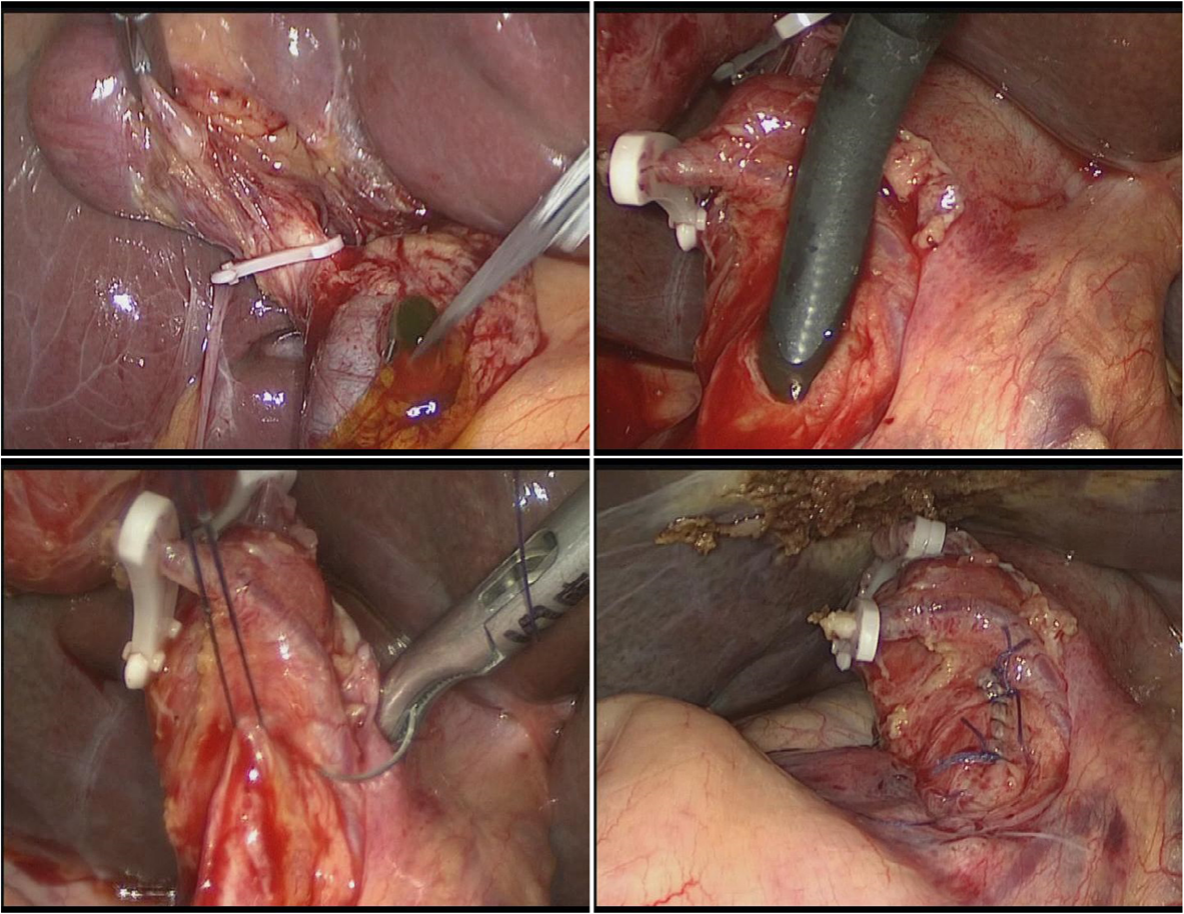
Intraoperative image of primary closure following LCBDE

After general anesthesia, the patient was placed in the supine position. The pressure of CO_2_ in abdominal cavity was maintained at 12 to 14 mmHg. Four trocars were placed in umbilical, medial epigastric, right anterior axillary line and right midclavicular area, respectively. The 10 mm trocar in right midclavicular area was placed for choledochoscope examination. After dissection of the gallbladder, the cystic duct was clipped with Hem-o-lock. Then the dissection continued to expose the cystic duct-CBD junction and the anterior wall of the CBD. The 1.0–1.5 cm longitudinal choledochotomy was made in the anterior surface of the CBD. After the choledochotomy, small stones were washed out by saline irrigation. Then, a flexible choledochoscope was routinely used to examine distal and proximal bile duct. The residual stones were extracted by using a stone basket. For big stones, electrohydraulic lithotripsy was used through the working channel of the choledochoscope. After complete remove the stones, the flexible choledochoscope was preformed to confirm the clearance of the intrahepatic/extrahepatic bile duct and the normal function of Oddi sphincter. Then, primary closure was performed with continuous or interrupted suture using Vicryl 3–0 or 4–0 suture line. Intraoperative cholangiography (IOC) was not performed (Fig. 1).

The definition of bile leakage was as follows: (1) for patients with drainage tube, bile was detected in the drainage for more than 3 days or the volume of drainage containing bile was more than 100 ml/day; (2) for patients without drainage tube, local or general peritonitis was detected and bile was found in ascites by puncture or reoperation.

### Possible risk factors

Possible risk factors for bile leakage after primary closure following LCBDE are divided into three groups: demographic factors, preoperative condition and surgical details.

Demographic factors: (1) age; (2) sex; (3) ASA score.

Preoperative condition: (1) serum leukocytes; (2) total bilirubin; (3) diameter of CBD; (4) No. of stones; (5) gallstone.

Surgical details: (1) operative time; (2) total blood loss; (3) method of suture; (4) length of choledochotomy; (5) surgeon’s experience.

### Statistics

The data is expressed as means ± SD or median (range). For pairwise comparisons of groups, we used the chi-square test or Fisher exact test for categorical variables and Student t test for quantitative variables. The logistic regression analysis was used to identify variables associated with risks for bile leakage after primary closure following LCBDE. All variables with univariable *p <* 0.1 were considered for the multivariable model. Results were presented as odds ratios (ORs) with 95% confidence intervals (CI). A *P* value <0.05 was considered statistically significant. Statistical analysis was performed with SPSS version 18.0 (SPSS Inc., Chicago, IL, USA).

## Results

### Patients

Between Feb. 2012 and Jun. 2016, 3109 patients with gallstones and 589 with CBD stones were treated in our hospital. Of the patients with choledocholithiasis, 376 patients received surgical therapy with 111 open and 265 laparoscopic procedures, respectively. In laparoscopic operation group, 8 patients converted to open surgery. The data about the patients who successfully received LCBDE was shown in Table 1. The mean diameter of CBD among patients with LCBDE procedure was 1.2 ± 0.3 cm. According to the MRCP or CT finding, there were 1.7 ± 1.3 stones detected in CBD. 82.8% (213/257) of the patients were complicated with gallstones. After LCBDE, 141 patients received primary closure and were included in this study. Among these cases, 92.2% (130/141) of patients were presented with abdominal pain and 49.6% (70/141) went to the emergency department. Obstructive jaundice was observed in 90 (64.5%) patients. In the same period, 23 patients with CBD stones received ERCP therapy in our hospital.

**Table 1:** Patients characters with CBD stones treated by LCBDE and primary closure after LCBDE

	LCBDE, *n =* 257	LCBDE + primary closure, *n =* 141
Male/Female	122/135	72/69
Age (years), mean ± SD	59.2 ± 15.3	58.9 ± 15.2
ASA score (I/II/III/IV)	41/143/55/18	22/79/32/8
Diameter of CBD (cm), mean ± SD	1.2 ± 0.3	1.1 ± 0.3
No. of stones, mean ± SD^a^	1.7 ± 1.3	1.7 ± 1.2
Length of choledochotomy (cm)	1.2 ± 0.6	1.3 ± 1.2
Primary closure/T-tube drainage	141/116	—
Postoperative bile leakage	20 (7.8%)	16 (11.3%)
Postoperative hemorrhage	8 (3.1%)	5 (3.5%)
Residual stones	10 (3.9%)	2 (1.4%)
Stone recurrence^b^	2 (0.8%)	2 (1.4%)
Bile duct stricture^b^	0	0
Duration of drainage placement (days)	4.0 ± 2.0	3.8 ± 2.0
Death	0	0

*ASA* American Society of Anesthesiologists

^a^according to the MRCP or CT finding, data available in 176 patients in LCBDE and 96 patients in primary closure group, respectively

^b^The median follow-up time was 34 (range 1–76) months

### Surgical results

No perioperative death occurred during the study period. The overall complication rate was 21.3 (30/141), and biliary-specific complication rate was 12.7% (18/141). According to Dindo-Clavien classification, most of the complications were as grade I, while one patient with uncontrolled bile peritonitis (grade IIIb) received reoperation (Table 2). Residual stones were found in 2(1.4%) patients by following CT and ultrasound examination.

**Table 2:** Complication after primary closure following LCBDE

Dindo-Clavien classification	Complication	No. of patients	Management
Grade I	Bile leakage Wound infection	10 7	Observation
Grade II	Bile leakage Fluid collection Pneumonia	5 4 2	Antibiotics
Grade III IIIa IIIb	Infected collection Bile leakage	1 1	Percutaneous drainage Reoperation

### Follow-up

The median follow-up time was 34 (range 1–76) months, stone recurrence was detected in 2 (1.4%) patients. Repeated LCBDE followed by T tube drainage was performed in these two cases. No bile duct stricture was found during the follow-up period.

### Fisk factors for bile leakage

Bile leakage occurred in 11.3%(16/141) of the patients with primary closure following LBCDE. Then, the patients were divided into two groups, bile leakage group and no bile leakage group. Most possible risk factors, such as age, ASA score, total bilirubin, operative time, total blood loss and et al were comparable between two groups. However, bile leakage occurred more frequently in patients with slender CBD (<1 *vs* ≥1 cm, 31.6% *vs* 7.0%, *p =* 0.037) and managed by inexperienced surgeons (initial 70 cases *vs* later cases, 17.1% *vs* 5.6%, *p =* 0.036) (Table 3).

**Table 3:** Possible risk factors for bile leakage after primary closure following LCBDE

	Bile leakage (*n =* 16)	No bile leakage (*n =* 125)	*P* value
Demographic factors			
Male/Female	7/9	65/60	0.534
Age (years) (≥70/<70)	4/12	29/96	0.873
ASA score (I + II/III + IV)	9/7	92/33	0.154
Preoperative condition			
Serum leukocytes (≥10/<10 × 10^9^/L)	2/14	23/102	0.564
Total bilirubin (≥23/<23 umol/L)	5/11	72/53	0.054
No. of stones (≥3/<3)	5/11	33/92	0.681
Diameter of CBD (<1/≥1 cm)	6/10	13/113	0.037
Gallstone (Yes/No)	12/4	97/28	0.799
Surgical details			
Operative time (≥2/<2 h)	2/14	28/97	0.793
Total blood loss (≥50/<50 ml)	1/15	23/102	0.250
Method of suture (continuous/interrupted)	13/3	106/19	0.713
Length of choledochotomy (≥1.5/<1.5 cm)	4/12	14/111	0.131
Surgeon’s experience (initial 70 cases/later cases)	12/4	58/67	0.036

*ASA* American Society of Anesthesiologists

The influences of age, sex, ASA score, serum leukocytes, total bilirubin, diameter of CBD, No. of stones, gallstone, operative time, total blood loss, method of suture, length of choledochotomy and surgeon’s experience on bile leakage after primary closure following LCBDE were summarized in Table 4. The slender CBD and inexperienced surgeons were associated with high risk of bile leakage in univariate analysis. Factors that affected bile leakage at the *p <* 0.10 level of significance by univariate analysis were included in a multivariate regression model. After adjusting for these variables, slender CBD [OR 95% CI, 3.799 (1.081–13.349), *p =* 0.04] and inexperienced surgeons [OR 95% CI, 4.228 (1.330–13.438), *p =* 0.03] were found to affect bile leakage.

**Table 4:** Results of Univariable and Multivariable Regression for bile leakage after primary closure following LCBDE

Risk factors	Univariable regression		Multivariable regression	
	OR (95% CI)	*P* value	OR (95% CI)	*P* value
Sex (male/Female)	0.718 (0.252–2.048)	0.53		
Age (years) (≥70/<70)	1.103 (0.331–3.683)	0.87		
ASA score (I + II/III + IV)	2.168 (0.748–6.289)	0.15		
Serum leukocytes (≥10/<10 × 10^9^/L)	0.634 (0.135–2.982)	0.56		
Total bilirubin (≥23/<23 umol/L)	0.335 (0.110–1.020)	0.05	0.587 (0.146–2.132)	0.23
No. of stones (≥3/<3)	1.267 (0.410–3.921)	0.68		
Diameter of CBD (<1/≥1 cm)	3.604 (1.092–11.898)	0.04	3.799 (1.081–13.349)	0.04
Gallstone (Yes/No)	0.871 (0.298–2.546)	0.80		
Operative time (≥2/<2 h)	0.838 (0.222–3.153)	0.79		
Total blood loss (≥50/<50 ml)	0.296 (0.037–2.353)	0.25		
Method of suture (continuous/ interrupted)	1.287 (0.335–4.951)	0.71		
Length of choledochotomy (≥1.5/<1.5 cm)	2.643 (0.749–9.324)	0.13		
Surgeon’s experience (initial 70 cases/later cases)	3.356 (1.026–10.974)	0.04	4.228 (1.330–13.438)	0.03

## Discussion

In the era of mini-invasive surgery, laparoscopic cholecystectomy (LC) has been the standard therapy for symptomatic gallstones. However, debate continues regarding the best treatment for managing cholecystocholedocholithiasis, and a consensus has not been reached [12, 13]. In clinical practice, three major procedures, LC + endoscopic retrograde cholangiopancreatography (ERCP)/endoscopic sphincterotomy (EST)/endoscopic papillary balloon dilation (EPBD), LC+ laparoscopic transcystic common bile duct exploration exploration (LTCBDE) and LCBDE were applied for treatment of cholecystocholedocholithiasis*.*

The role of ERCP in diagnosis of CBD stones has been replaced by MRCP, however, it is widely used to remove CBD stones in one-stage or staged procedures. EST is associated with serious short-term complications, including bleeding, post-ERCP pancreatitis (PEP), and perforation of the digestive tract. In addition, EST may increase the incidence of long-term complications such as biliary infection due to the dysfunction of the Oddi’s sphincter after the procedure [14–16]. In order to preserve (at least partly) the function of the sphincter of Oddi and avoid post-EST bleeding, EPBD is more and more used in treatment of CBD stones. Actually, choledocholithiasis is the only indication for EPBD reported in large controlled series. Unfortunately, the recent RCTs failed to demonstrate the advantages of EPBD over EST in terms of post-operative complications including PEP, bleeding and perforation [17, 18]. The rate of complete stone removal and utilization of endoscopic mechanical lithotripsy in EST group was comparable with EPBD group [19]. Meta-analyses also showed similar efficacy and overall safety (less bleeding but more PEP in EPBD) between the two treatments [20]. In patients with cholecystocholedocholithiasis, ERCP with stone extraction might be performed selectively before, during or after cholecystectomy. However, as discussed by Costi et al., there were several limitations about ERCP therapy [21]. Firstly, performing ERCP before surgery raised questions regarding patient selection, but no consensus has reached even in the recent guidelines from US and Europe [13, 22]. Secondly, performing ERCP contextually to LC implied organizational problems concerning the availability of an endoscopist in the operating theater whenever needed. Finally, performing ERCP after surgery would raise the dilemma of managing CBD stones whenever ERCP failed to retrieve them because a third procedure would then be needed.

LTCBDE was firstly preformed by Stoker in 1995 [23]. Recent report from Zhu demonstrated that in their 708 patients, LTCBDE could be successfully applied in more than 90% patients and with high clearance rate (common bile duct stones residual in 13 patients) and low complication rate (postoperative complications in 27 patients). So they conclude that LTCBDE with or without microincision and/or lithotripsy is a safe and effective approach in treatment of CBD stones [24]. However, the ability to remove stones is restricted by the diameter of cystic duct, even with application of electrohydraulic lithotripsy. In patients with many CBD stones or slender cystic duct, LTCBDE may lead to the high rate of stones retained [25, 26]. To avoid these limitations, LCBDE was used to access to a common bile duct without causing damage to the biliary sphincter and also with the high clearance rate. Several RCTs have revealed the effectiveness and safety of LCBDE comparing with LC+ ERCP/EST. The clearance rate form these studies reached 88% to 100%, which was as effective as EST procedure, without increased in the morbidity and mortality rates. Moreover, LCBDE might result in shorter hospitalizations and lower costs [27–29]. The meta-analysis also revealed that no statistically significant difference in stone clearance [risk ratios (RR) = -0.10, 95% confidence intervals (CI): -0.24 to 0.04, *p =* 0.17], postoperative morbidity (RR = 0.79, 95% CI: 0.58 to 1.10, *p =* 0.16), mortality (RR = 2.19, 95% CI: 0.33 to 14.67, *p =* 0.42), and length of hospital stay (MD = 0.99, 95% CI: -1.59 to 3.57, *p =* 0.45) [30], which was in accordance with the cochrane systematic review [31]. However, in the recent RCT comparing single-stage LCBDE and cholecystectomy versus two-stage endoscopic stone extraction followed by laparoscopic cholecystectomy, significantly greater number of patients with severe complications were detected in staged group (≥grade III: 11.7% vs 0%, *p =* 0.0004), although the overall morbidity was comparable between the two groups (23.8% vs. 22.6%, *p =* 1.0) [32]. In our cases series (*n =* 265), the technique successful rate was 97.0% (257/265), and only 10 (3.9%) patients was observed with residual stones. Bile leakage was most common complication (7.8%, 20/257), followed by residual stones and postoperative hemorrhage (3.1%, 8/257). In our hospital, ERCP was not the first choice in treatment of CBD stones. The reasons were as follows: (1) one-stage operation was as safe and efficacy as staged approach, even in patients with high medical risk [27], but an experienced endoscopist might not be available when needed; (2) ERCP was a costly procedure in China; (3) possibility of serious complications and the bad relationship between doctors and patients now [33] hindered the endoscopist’s enthusiasm, especially when there was no strong evidence proving the advantage of ERCP procedure in treatment of CBD stones; (4) surgeons with advanced laparoscopic skills were easy to obtain in our hospital. So, we preferred LCBDE + LC to treat cholecystocholedocholithiasis because of the efficacy, safety and low cost*.*

After LCBDE, both T-tube drainage and primary closure are widely used in clinical practices. Several comparative studies and meta-analysis have demonstrated that primary closure is at least as effective and safe as T-tube drainage [8, 34, 35], even in emergency operation [36] and elderly patients [37]. Long-term outcome also shown that primary closure after LCBDE had a low incidence of recurrent stones, and no biliary strictures [5]. Thus, primary closure after LCBDE with choledochoscopy is considered to be a safe and effective alternative to T-tube drainage. However, primary closure after LCBDE is still a technique demanding procedure and bile leakage is the most frequently postoperative complication.

The incidence of bile leakage after primary closure following LCBDE varied dramatically [38–40]. It might be attributed to the different definition of bile leakage after biliary surgery among centers. Only few papers explained the definition of bile leakage they used [41]. In our hospital, bile leakage occurred in about 11.3% of patients with primary closure. Although most of them (62.5, 10/16) was mild (Dindo-Clavien GradeI), it seems higher than previously reported. We believed it might be resulted from the stricter criteria we used when comparing with the ISGLS definition [42].

Bile leakage negatively influences the postoperative recovery and patient might need additional imaging study and even reoperation. However, the risk factors for bile leakage after primary closure following LCBDE has not been fully discussed. In our study, we found that the surgeon’s experience was the most important risk factor for bile leakage [OR 95% CI, 4.228 (1.330–13.438), *p =* 0.03]. In experienced hands, the incidence of bile leakage decreased significantly form 15.7% to 7.0% (*p =* 0.04). The data form LC, LCBDE and LCTBDE also revealed the importance of surgeon’s experience for decreasing the complication rate [24, 26, 43]. Another factor affecting the incidence of bile leakage was the diameter of CBD, which was in accordance with Hua’s study [40]. In patients with slender CBD, bile leakage occurred more frequently (<1 *vs* ≥1 cm, 31.6% *vs* 7.0%, *p =* 0.04). Possible reasons for this tendency were as follows: (1) the wall was thin in patients with slender CBD, and the bile could leak from the needle pinprick; (2) when suturing, surgeons might stitch too little tissue because of the fear of CBD stricture; (3) after suturing, transient stenosis of CBD might occur due to the tissue edema, and pressure increased within the biliary tree, then bile leakage occurred. So, in these cases, transcystic biliary drainage should be considered to reduce the pressure within the biliary tree.

In our cases series, one patient received reoperation due to the uncontrolled bile peritonitis. We found intraoperatively that the first suture was too far away from the superior edge of bile duct incision. So in the following cases, we usually preform the first suture above the superior edge of incision to avoid bile leakage resulting from excessive traction in following sutures. Absorbable sutures were used in our hospital to avoid CBD stricture and stone recurrence. In the follow-up period (median 34 moths, range 1–76 months), no CBD stricture was observed and 2 patients experienced stone recurrence.

Despite the limitations of retrospective study design and limited case volume, it was the initial try to detect the risk factors for bile leakage after primary closure following LCBDE. We found that slender CBD and inexperienced surgeons were associated with high bile leakage risk independently in primary closure after LCBDE. However, we believed that primary closure after LCBDE was safe in selected patients preformed by experienced hands.

## Conclusion

Slender CBD and inexperienced surgeons were the high risk factors for bile leakage after primary closure following LCBDE.
